# Multi-Scale Meteorological Impact on PM_2.5_ Pollution in Tangshan, Northern China

**DOI:** 10.3390/toxics12090685

**Published:** 2024-09-22

**Authors:** Qian Liang, Xinxuan Zhang, Yucong Miao, Shuhua Liu

**Affiliations:** 1 State Key Laboratory of Severe Weather & Key Laboratory of Atmospheric Chemistry of CMA, Chinese Academy of Meteorological Sciences, Beijing 100081, China; liangqian456@126.com (Q.L.); zhangxx199908@163.com (X.Z.); 2 Changzhi Meteorological Bureau, Changzhi 046000, China; 3 Department of Atmospheric and Oceanic Sciences, School of Physics, Peking University, Beijing 100871, China

**Keywords:** PM_2.5_ pollution, synoptic patterns, regional transport, planetary boundary layer

## Abstract

Tangshan, a major industrial and agricultural center in northern China, frequently experiences significant PM_2.5_ pollution events during winter, impacting its large population. These pollution episodes are influenced by multi–scale meteorological processes, though the complex mechanisms remain not fully understood. This study integrates surface PM_2.5_ concentration data, ground-based and upper–air meteorological observations, and ERA5 reanalysis data from 2015 to 2019 to explore the interactions between local planetary boundary layer (PBL) structures and large-scale atmospheric processes driving PM_2.5_ pollution in Tangshan. The results indicate that seasonal variations in PM_2.5_ pollution levels are closely linked to changes in PBL thermal stability. During winter, day–to–day increases in PM_2.5_ concentrations are often tied to atmospheric warming above 1500 m, as enhanced thermal inversions and reduced PBL heights lead to pollutant accumulation. Regionally, this aloft warming is driven by a high-pressure system at 850 hPa over the southern North China Plain, accompanied by prevailing southwesterly winds. Additionally, southwesterly winds within the PBL can transport pollutants from the adjacent Beijing–Tianjin–Hebei region to Tangshan, worsening pollution. Simulations from the chemical transport model indicate that regional pollutant transport can contribute to approximately half of the near-surface PM_2.5_ concentration under the unfavorable synoptic conditions. These findings underscore the importance of multi-scale meteorology in predicting and mitigating severe wintertime PM_2.5_ pollution in Tangshan and surrounding regions.

## 1. Introduction

Despite the implementation of stringent emission reduction measures by the Chinese government since 2013, which have led to steady improvements in air quality, severe PM_2.5_ pollution events continue to occur during autumn and winter [1,2]. These pollution episodes pose significant public health risks, including impaired visibility, increased incidence of cardiopulmonary diseases, and premature mortality [3,4].

PM_2.5_ pollution is influenced not only by anthropogenic emissions but also by multi-scale meteorological conditions [5,6,7]. Locally, pollutants emitted at the surface undergo processes such as diffusion, transport, deposition, and chemical transformation, all of which are regulated by the planetary boundary layer (PBL) [8,9]. The depth of the PBL plays a crucial role in determining the vertical dispersion capacity of these pollutants [10,11,12]. Additionally, winds within the PBL are key determinants of the horizontal transport of pollutants, influencing both direction and distance [13,14]. The formation and evolution of PBL structures are often associated with regional synoptic patterns. Slow–moving or stationary high–pressure systems induce sinking airflows and stable conditions, which dynamically suppress the development of the PBL and result in the accumulation of pollutants near the ground [15,16]. Moreover, synoptic warm advection above the PBL can strengthen thermal inversions, inhibiting PBL development and exacerbating ground-level pollution [17,18].

Additionally, the regional transport of pollutants is influenced by the evolution of synoptic systems [19,20,21]. For example, a cold front accompanied by strong northwest winds can help disperse local pollutants, but may also transport pollutants from the North China Plain to the downstream Yangtze River Delta region [22]. Similarly, under the influence of a high–pressure system over North China, aerosols emitted from the Beijing–Tianjin–Hebei region can be transported to Shenyang by westerly and southwesterly synoptic winds [23].

**Figure 1:**
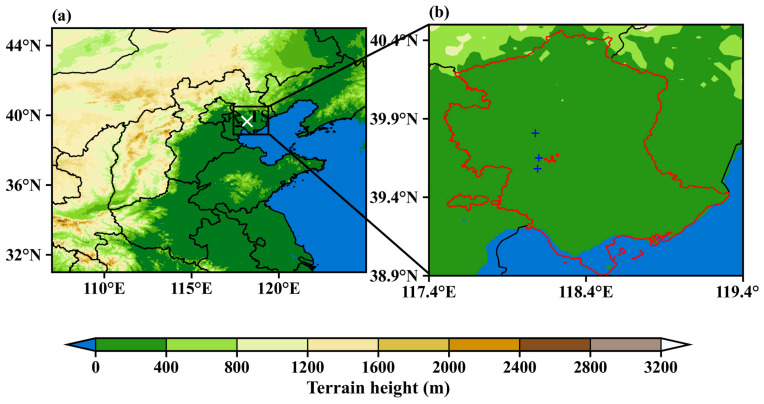
Maps displaying the terrain height for (**a**) North China and (**b**) the Tangshan region. In (**a**), the radiosonde station is indicated by a white cross. In (**b**), the air quality monitoring stations are represented by red dots, while the ground–level meteorological stations are marked with blue crosses. The red contour in (**b**) delineates the boundary of Tangshan.

Tangshan, located in the northeastern Beijing–Tianjin–Hebei region and facing the Bohai Sea (Figure 1), has a population of approximately 7 million. The city frequently experiences land–sea breezes, which can influence the distribution and accumulation of pollutants [24]. Furthermore, as a traditional resource-based industrial city, Tangshan’s economy relies heavily on industries such as steel, coal, cement, and chemicals, making it the top gross domestic product contributor in Hebei province [25]. However, these industries also contribute to frequent and severe PM_2.5_ pollution events [26]. Following rapid environmental deterioration in the past, China implemented its strictest environmental law in 2015, leading to significant reductions in pollutant emissions [27]. While extensive research has been conducted on PM_2.5_ pollution in megacities in northern China like Beijing and Tianjin [28,29], Tangshan has received comparatively less attention. Unlike the diversified industrial structures of Beijing and Tianjin, Tangshan has a heavy industrial focus, with steel production, cement manufacturing, and coal consumption being major contributors to its emissions. This industrial profile, combined with its coastal location and unique meteorological patterns, creates distinct challenges for air quality management. Therefore, this study focuses on the impacts of multi-scale meteorological processes on PM_2.5_ pollution in Tangshan, utilizing multi–source environmental and meteorological data from 2015 to 2019.

## 2. Materials and Methods

In this study, we collected hourly meteorological data for Tangshan from 2015 to 2019 from three ground-level meteorological stations (marked by blue crosses in Figure 1b). Given that heavy pollution episodes typically occur on days without precipitation, we excluded data from days with precipitation in Tangshan (i.e., 24 h accumulated precipitation exceeding 0.5 mm at any of the three stations). Additionally, hourly PM_2.5_ measurements from 2015 to 2019 were obtained from six air quality monitoring stations (marked by red dots in Figure 1b), which are operated by the China National Environmental Monitoring Center (CNEMC). We calculated daily PM_2.5_ concentrations for each monitoring station and then averaged these values across all stations to derive daily mean concentrations. For pollutant emission data, we used the Multi-resolution Emission Inventory for China (MEIC) developed by Tsinghua University, with a horizontal resolution of 0.25° for the winters from 2015 to 2019, which is widely utilized in air pollution prediction and management [30,31,32].

For upper-level meteorological data, we utilized observations from a radiosonde station (marked by a white cross in Figure 1a), which conducted soundings twice daily at 08:00 and 20:00 local time (LT = UTC + 8 h). These soundings provide profiles of temperature, pressure, relative humidity, and wind, with a fine vertical resolution of approximately 10 m [9]. The boundary layer height (BLH) in Tangshan was estimated from sounding data using the bulk Richardson method [33], with the BLH defined as the lowest level where the Richardson number exceeds 0.25 [18]. To supplement the twice–daily soundings, we analyzed the fifth-generation atmospheric reanalysis produced by the European Center for Medium-Range Weather Forecasts (ERA5) [34,35,36], which assimilates both surface and upper-level observations. ERA5 provides data with a horizontal resolution of 0.25° and a temporal resolution of 1 h, allowing for a more detailed analysis of atmospheric conditions across time and space. We also calculated the thermal difference of the potential temperature (TD), which is analogous to lower tropospheric stability and can be used to quantify the PBL thermal stability [37,38].

To objectively analyze the relationships between synoptic patterns, PBL structures, and PM_2.5_ pollution in Tangshan, we performed a T–mode principal component analysis (T–PCA) on the fields of pressure or geopotential height over North China (108–128° E, 29–49° N) at 14:00 LT during the winter, deriving from the ERA5 data. T–PCA is an analytical method used to identify the most representative spatial synoptic patterns and their temporal occurrences [39]. It has been widely employed to explore the linkages between synoptic conditions and air pollution in China, due to its ability to effectively handle high-dimensional data, reduce noise, and account for temporal dynamics, making it more robust than methods like K–means clustering or Self–Organizing Maps [39,40,41,42]. After identifying dominant synoptic patterns, we analyzed the spatial distribution of mean PM_2.5_ concentrations during all instances when these patterns occurred, using the Chinese Air Quality Reanalysis (CAQRA) dataset, which assimilates the CNEMC surface observations through an ensemble Kalman filter and the Nested Air Quality Prediction Modeling System [43].

Additionally, we used the Hybrid Single Particle Lagrangian Integrated Trajectory (HYSPLIT) model [44] to simulate 24 h backward trajectories of air masses, helping to identify pollutant transport pathways. For each day, the trajectory end time was set to 20:00 LT, with the endpoint located in Tangshan (118.18° E, 39.64° N, 100 m above ground level). We applied cluster analysis [45] to group the air mass trajectories based on their speed and direction, intuitively determining the source regions and transport distances of dominant air masses. Based on the simulated backward trajectories, we calculated the Potential Source Contribution Function (PSCF) for the PM_2.5_ pollution threshold in Tangshan [46,47]. The PSCF was computed at a resolution of 0.5° × 0.5°, dividing the study area into i × j grids, including Tangshan and its surrounding areas. The PSCF value for each grid cell was determined by m_ij_/n_ij_, where n_ij_ represents the number of trajectory endpoints within the grid cell and m_ij_ represents the number of “polluted” trajectory endpoints in the same grid cell. The “polluted” trajectory was determined based on the top 20% of daily concentrations during the winter season. To account for uncertainties in grids with low n_ij_ values, we applied a weighting factor W_ij_ to reduce result uncertainties [48]. The Weighted Potential Source Contribution Function (WPSCF) is defined as follows:

(1) $${WPSCF}_{ij}=\frac{m_{ij}}{n_{ij}}W_{ij}$$

(2) $$W_{ij}=\left\{\begin{array}{c} 1.00\;\;\;\;\;\;\;\;\;\;\;\;\;\;\;\;\;\;\;\;\;\;\;\;3n_{ave}<n_{ij}\\ 0.7\;\;\;\;\;\;1.5n_{ave}<n_{ij}\leq \;3n_{ave}\\ 0.42\;\;\;\;\;n_{ave}<n_{ij}\leq \;1.5n_{ave}\\ 0.05\;\;\;\;\;\;\;\;\;\;\;\;\;\;\;\;\;\;\;\;\;\;\;\;\;n_{ij}\leq \;n_{ave} \end{array}\right.$$

where $n_{ave}$ represents the average number of trajectory endpoints per grid cell.

After identifying the dominant synoptic pattern and relevant transport pathways, we utilized the Weather Research and Forecasting model coupled with Chemistry (WRF-Chem version 3.9.1) to simulate the spatial distribution of PM_2.5_ concentrations in Tangshan during a typical pollution episode on 3 December 2016. The simulation domain was centered on the Beijing–Tianjin–Hebei region, with a horizontal resolution of 13 km, covering an area from 107 to 128° E in longitude and 31.5 to 46° N in latitude. The physics parameterization schemes applied in the WRF–Chem simulation included the Noah land surface model [49], the YSU boundary layer scheme [50], the RRTMG long–/short–wave radiation scheme [51], and the RADM2-MADE/SORGAM chemical mechanism [52,53,54]. The simulation was initialized at 20:00 LT on December 1 and ran for 52 h, concluding at 00:00 LT on December 4. The first 28 h were treated as a spin-up period to ensure stable conditions. Anthropogenic emissions were set using the MEIC, while initial and boundary conditions for meteorological variables were derived from the ERA5 reanalysis dataset. This configuration is referred to as the BASE run. To quantify the contributions of local emissions and regional transport to PM_2.5_ pollution in Tangshan, we conducted a sensitivity simulation by zeroing out the local anthropogenic emissions in Tangshan (117.4–119.4° E, 38.9–40.5° N). This sensitivity simulation is referred to as the blank (BLK) run.

## 3. Results and Discussion

### 3.1. Seasonal Variations of Pollution and Their Association with PBL Thermal Structures

**Figure 2:**
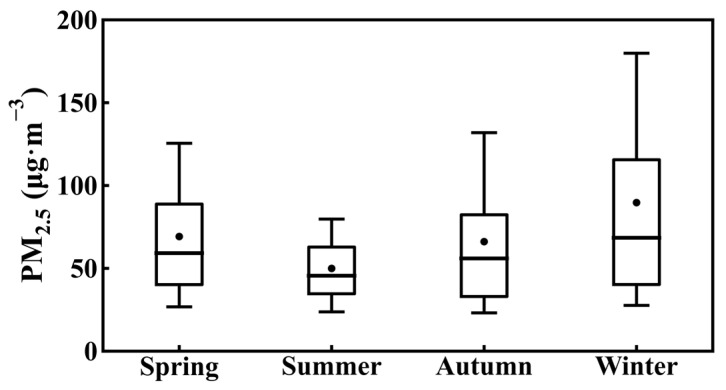
Seasonal variations of observed PM_2.5_ concentrations in Tangshan, based on ground-level measurements from 2015 to 2019. In the boxplot, the central box represents the interquartile range (25th to 75th percentile), the vertical line extends from the 10th to 90th percentile, the solid line within the box denotes the median, and the dot represents the mean value.

**Figure 3:**
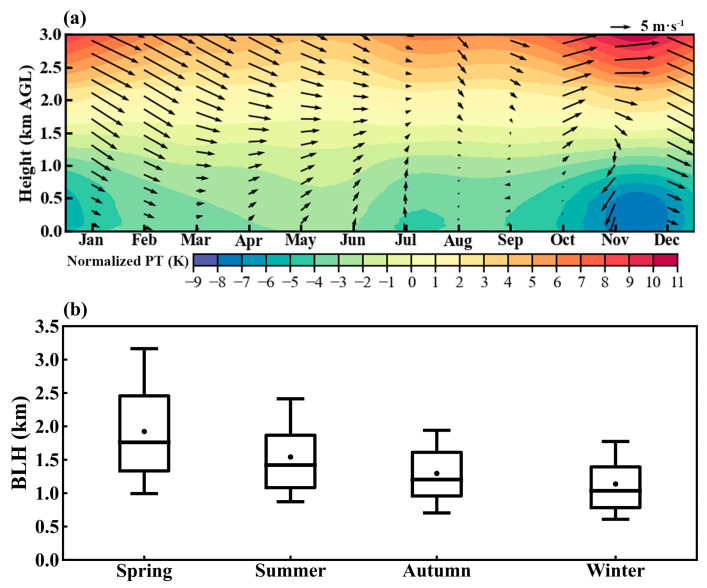
(**a**) Time–height sections of monthly normalized potential temperature (PT), with overlaid horizontal wind vectors (black arrows), and (**b**) seasonal variations of boundary layer height (BLH) at 14:00 LT in Tangshan, derived from the ERA5 dataset. In (**a**), the PT profile for each month is normalized by subtracting the mean value below 3000 m. In (**b**), the central box represents the interquartile range (25th to 75th percentile), the vertical line extends from the 10th to 90th percentile, the solid line within the box denotes the median, and the dot represents the mean value.

The annual average PM_2.5_ concentration in Tangshan from 2015 to 2019 was 68.5 μg m^−3^, significantly exceeding the Chinese national standard of 35 μg m^−3^, with distinct seasonal variations (Figure 2). The lowest average PM_2.5_ concentration was recorded in summer at 50 μg m^−3^, followed by spring and autumn at 69 μg m^−3^ and 66 μg m^−3^, respectively, with winter showing the highest concentration at 90 μg m^−3^. A large interquartile range in PM_2.5_ concentrations indicates diverse sources or varying dispersion patterns, particularly noticeable in winter (Figure 2). The severe winter PM_2.5_ pollution is linked to both seasonal increases in anthropogenic emissions (Figure S1) and strong thermal stability in the lower troposphere (Figure 3) [55]. Using ERA5 reanalysis data, we calculated monthly normalized potential temperature profiles by subtracting the mean potential temperature from the surface to 3000 m for each month (Figure 3), revealing a steeper potential temperature gradient between the upper atmosphere (3000 m) and the surface during winter compared to other seasons (Figure 3a). This gradient suggests stronger thermal stability in the lower troposphere during winter, leading to a lower BLH, with an average of 1140 m at 14:00 LT (Figure 3b). In contrast, the average BLH at 14:00 LT is highest in spring, exceeding 1900 m, followed by 1540 m in summer and 1300 m in autumn (Figure 3b). The thermal structure of the lower troposphere and PBL during winter is more conducive to the formation and persistence of severe PM_2.5_ pollution compared to other seasons [56,57,58]. Similar seasonal variations in lower tropospheric thermal structures were also observed in the sounding data (Figure S2), demonstrating good consistency between the sounding data and ERA5. In the following sections, we will delve into the heavy pollution events in winter and the underlying physical mechanisms.

### 3.2. Day–to–Day Variations in the PBL Structure and Their Impacts on Pollution

**Figure 4:**
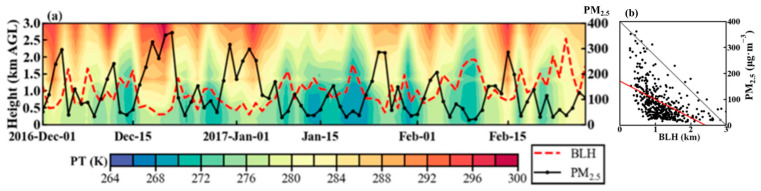
(**a**) Time–height sections of PT from 1 December 2016 to 28 February 2017, overlaid with BLH (marked by a red dashed line) at 14:00 LT and daily mean PM_2.5_ concentration (marked by a black dotted line), and (**b**) scatter plot comparing BLH at 14:00 LT and daily mean PM_2.5_ concentration for all winters from 2015 to 2019. In (**b**), the gray diagonal line represents a 1:1 reference line, and the red line shows the least squares fitting line. The PBL data were derived from the ERA5 dataset, and the PM_2.5_ concentration is based on measurements.

This section analyzes the day–to–day variations in PM_2.5_ pollution and PBL structure in Tangshan during winter. Using ERA5 reanalysis data, Figure 4a illustrates the daily changes in potential temperature profiles at 14:00 LT alongside the corresponding PM_2.5_ concentrations in Tangshan from December 2016 to February 2017. Severe PM_2.5_ pollution episodes are frequently associated with significant warming above 1500 m and a reduction in the afternoon BLH (Figure 4a). For example, from December 15 to 20, PM_2.5_ concentrations in Tangshan surged from 53 μg m^−3^ to 352 μg m^−3^, while the TD between 3000 m and 100 m increased from 3.4 K to 13 K, and the afternoon BLH dropped to approximately 400 m (Figure 4a). These pronounced changes in the PBL’s thermodynamic structure were also observed in the radiosonde measurements (Figure S3). Enhanced thermal stability in the lower troposphere limits the vertical extension of the PBL, limiting the vertical dispersion of PM_2.5_ and contributing to pollution events (Figure 4b). Similar relationships regarding wintertime PM_2.5_ pollution also can be found in other winter months from 2015 to 2019.

**Figure 5:**
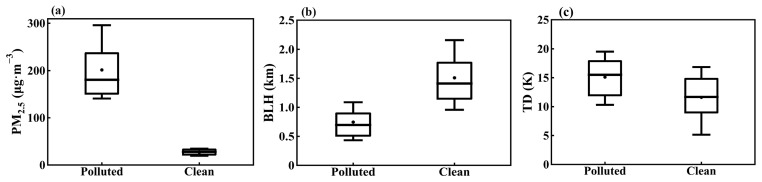
Differences between polluted and clean days in (**a**) daily mean PM_2.5_ concentrations, (**b**) BLH, and (**c**) the thermal difference (TD) between surface and 3000 m at 14:00 LT. In the boxplot, the central box represents the interquartile range (25th to 75th percentile), the vertical line extends from the 10th to 90th percentile, the solid line within the box denotes the median, and the dot represents the mean value. Results are calculated from PM_2.5_ measurements and ERA5 data in Tangshan during the winters of 2015–2019.

Following established studies on the relationship between PM_2.5_ pollution and meteorology [59,60], days with average daily PM_2.5_ concentrations in the upper 20th percentile were categorized as pollution days, while those in the lower 20th percentile were categorized as clean days. Pollution days were defined by a PM_2.5_ concentration threshold of 134 μg m^−3^, and clean days were defined at 36 μg m^−3^ (Figure 5a). On pollution days, the average afternoon BLH was 750 m, significantly lower than the 1510 m observed on clean days (Figure 5b). The TD on pollution days was 15.1 K, notably higher than the 11.5 K observed on clean days (Figure 5c).

**Figure 6:**
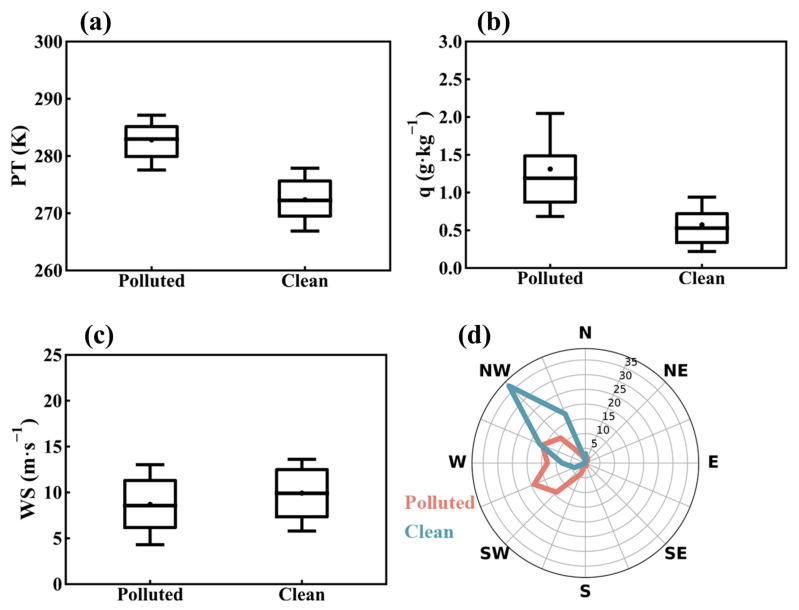
Differences between polluted and clean days in (from left to right) (**a**) PT, (**b**) specific humidity (q), (**c**) wind speed (WS), and (**d**) wind direction at 850 hPa in Tangshan during the winters of 2015–2019. The data were derived from the ERA5 dataset at 14:00 LT. In the boxplot, the central box represents the interquartile range (25th to 75th percentile), the vertical line extends from the 10th to 90th percentile, the solid line within the box denotes the median, and the dot represents the mean value.

**Figure 7:**
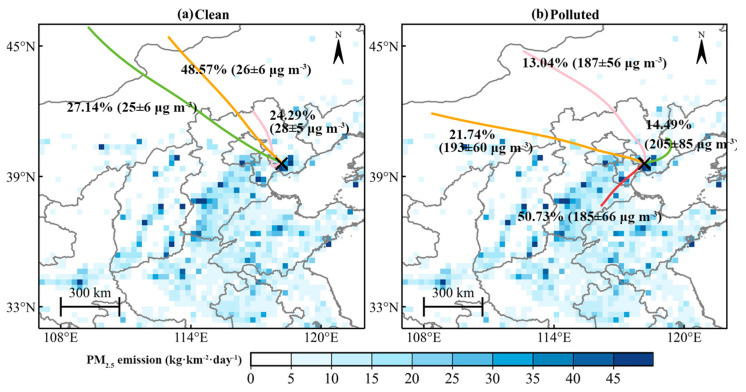
Spatial distribution of MEIC PM_2.5_ emissions in winter, overlaid with 24 h backward trajectory clustering for (**a**) clean days and (**b**) polluted days from 2015 to 2019. The mean and standard deviation of PM_2.5_ concentrations are indicated under each trajectory. The endpoint of backward trajectories is denoted by a black cross in each panel. The radiosonde station of Tangshan is marked with a black cross.

**Figure 8:**
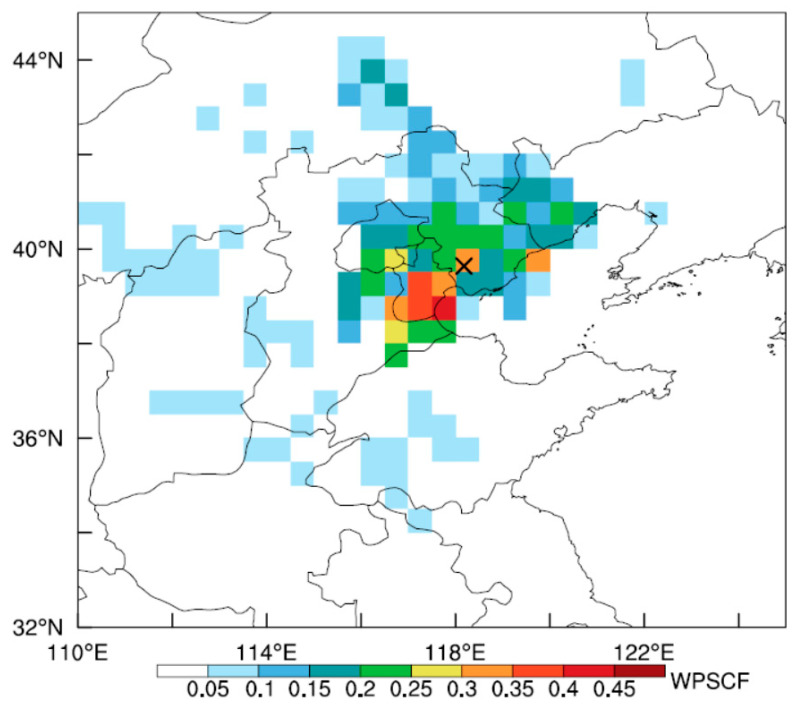
Maps of the Weighted Potential Source Contribution Function (WPSCF) for the top 20% of PM_2.5_ pollution in Tangshan, based on the concentration threshold of 134 μg m^−3^. The endpoint of the backward trajectories is indicated by a black cross.

At the 925 hPa, 850 hPa, and 700 hPa levels, northwest winds dominated on clean days, contrasting with southwest winds on pollution days (Figure S4). Northwest winds typically bring colder, drier air masses to Tangshan, whereas southwest winds are associated with warmer, more humid polluted air masses (Figure 6). The range of variation in specific humidity and wind direction on polluted days is larger than that on clean days (Figure 6). Under polluted conditions, relative humidity is higher compared to clean conditions. Additionally, wind speeds are slightly lower during polluted periods. These variations in humidity and wind speed significantly influence PM_2.5_ levels. Higher humidity enhances the formation and persistence of particulate matter by increasing aerosol water content and facilitating chemical reactions [61,62]. Meanwhile, calm wind conditions allow pollutants to accumulate, further contributing to elevated PM_2.5_ concentrations. The influence of these differing prevailing wind directions is further evident from the HYSPLIT backward trajectories (Figure 7). The air masses reaching Tangshan on pollution days primarily originated from the southwest, west, northwest, and northeast (Figure 7b). Long–distance air masses from the northwest and northeast pass through relatively drier areas such as Inner Mongolia (Figure 7b). Short–distance air masses from the southwest direction, accounting for 50.73% of the trajectories, passed through high-emission regions such as Hebei and Tianjin (Figure 7b). The concentration threshold of 134 μg m^−3^, identified for pollution days during Tangshan’s winter, was used to derive the potential PM_2.5_ source areas (Figure 8). The WPSCF value for Tangshan is 0.3, with higher values exceeding 0.3 observed in the western coastal region of the Bohai Sea (Figure 8), particularly in Tianjin. This aligns with the identified short-distance transport pathway (Figure 7b), indicating that regional pollutant transport from nearby areas significantly contributes to Tangshan’s pollution.

### 3.3. Impact of Synoptic Patterns on the PBL and Pollution

**Figure 9:**
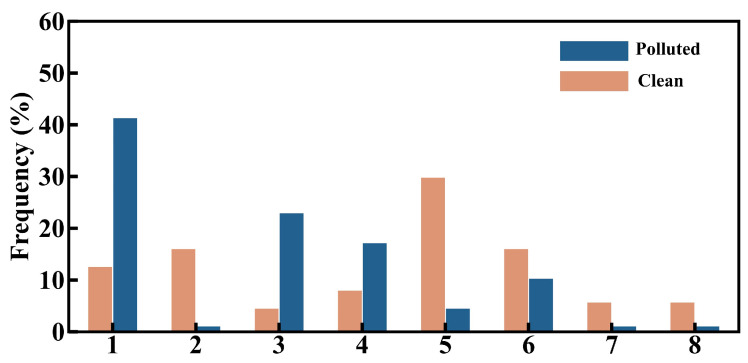
The occurrence frequency of polluted days (top 20% concentration, orange bars) and clean days (bottom 20% concentration, blue bars) under different classified synoptic types.

**Figure 10:**
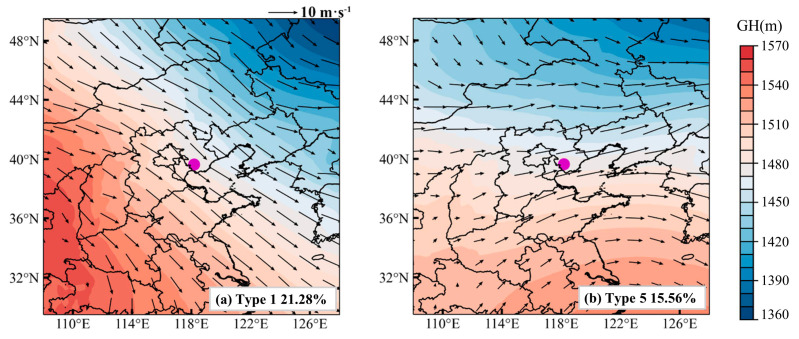
The 850 hPa geopotential height (GH) fields (color shading) of (**a**) Type 1 and (**b**) Type 5 identified using the T–PCA method, overlaid with wind vector fields (black arrows). The occurrence frequency (%) is indicated at the bottom of each panel, and the radiosonde station of Tangshan is marked with a purple dot.

**Figure 11:**
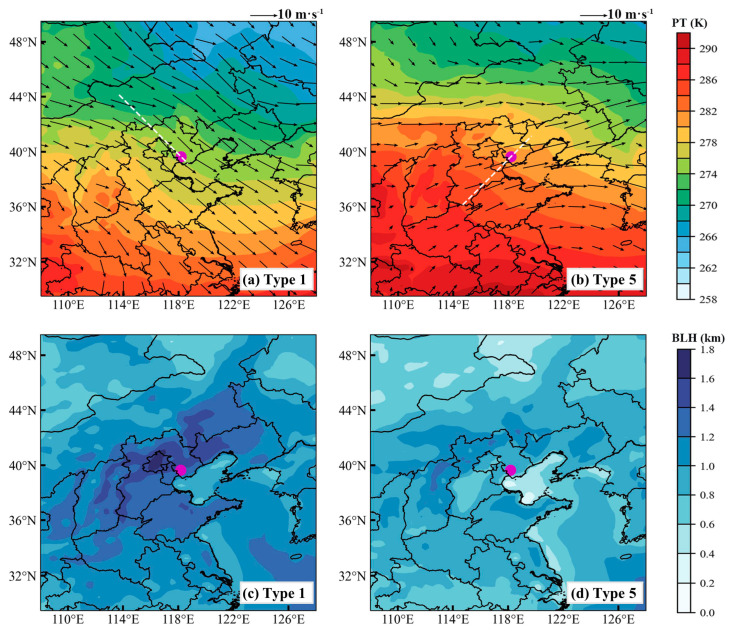
Spatial distributions of (**a**,**b**) 850 hPa PT overlaid with horizontal wind vector fields (black arrows) and (**c**,**d**) averaged BLH, under the influence of synoptic Type 1 (left panels) and synoptic Type 5 (right panels). The white dashed lines indicate the locations of cross sections shown in Figure 12. The radiosonde station of Tangshan is marked with a purple dot. The data were derived from the ERA5 dataset at 14:00 LT.

**Figure 12:**
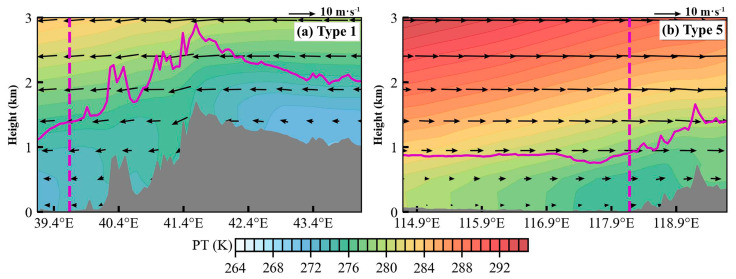
Vertical cross sections of PT and wind along the prevailing wind direction, cutting through Tangshan under of (**a**) synoptic Type 1 and (**b**) synoptic Type 5. The black arrows represent the 3D wind field projected onto the cross–sections, with vertical velocity multiplied by a factor of 20. The location of Tangshan is marked by the purple dashed line, and the BLH is indicated by the purple solid line in each panel. The data were derived from the ERA5 dataset at 14:00 LT.

To identify the dominant synoptic patterns associated with pollution and clean days in Tangshan, we applied the T–PCA method to analyze the geopotential height field at 850 hPa, identifying eight distinct synoptic patterns (Figure S5). Among these, Type 1 is the predominant pattern on clean days, while Type 5 is most frequent on pollution days (Figure 9). Type 1 is characterized by a southwest–to–northeast pressure gradient at 850 hPa over North China (Figure 10a), resulting in prevailing northwest winds in Tangshan (Figure 11a) and cold advection above 1000 m. In contrast, Type 5 is associated with a north–south pressure gradient over North China (Figure 10b), leading to prevailing southwest winds in Tangshan (Figure 11b) and warm advection from the southern North China Plain above 1000 m (Figure 12b). This warm air advection strengthens the thermal inversion layer above the PBL, suppressing its daytime development and reducing its depth (Figure 11d). Under Type 5, the average afternoon BLH in Tangshan is only 894 m, compared to over 1300 m under Type 1 (Table S1). The reduced BLH limits the vertical dispersion of pollutants, leading to the accumulation of surface-emitted pollutants near the ground [8].

**Figure 13:**
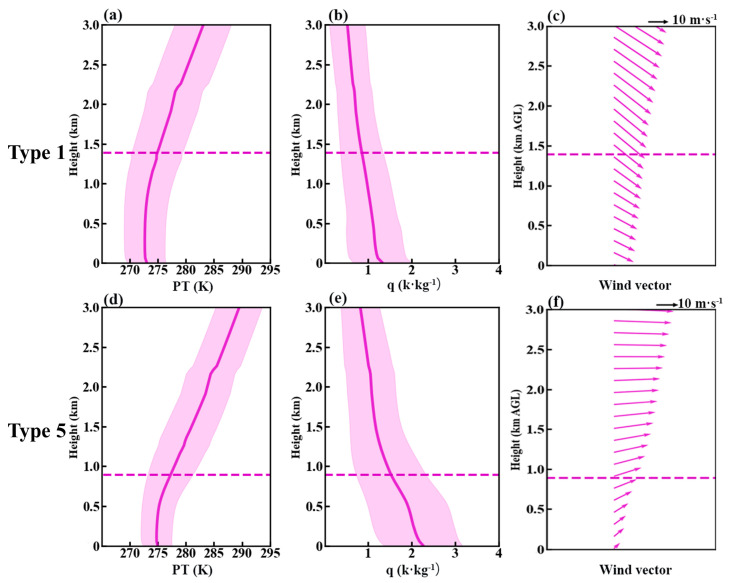
Vertical profiles of (**a**,**d**) PT (mean ± standard deviation), (**b**,**e**) q (mean ± standard deviation), and (**c**,**f**) horizontal wind vector (purple arrows) under polluted days in synoptic Type 5 and clean days in synoptic Type 1 at 14:00 LT in Tangshan. The purple dashed lines indicate the mean BLH for each type. The data were derived from the ERA5 dataset.

**Figure 14:**
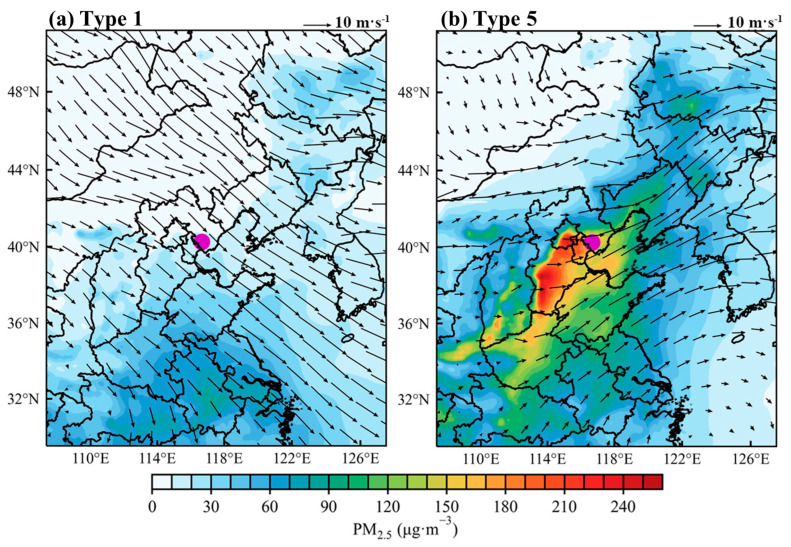
Averaged near–surface PM_2.5_ concentrations overlaid with 850 hPa wind vector fields (black arrows) during (**a**) clean days under synoptic Type 1 and (**b**) polluted days under synoptic Type 5. The radiosonde station of Tangshan is marked with a purple dot. PM_2.5_ concentration data were derived from the CAQRA dataset, and meteorological data were from ERA5.

**Figure 15:**
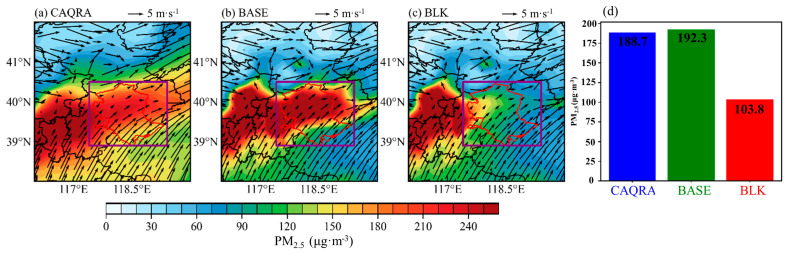
Spatial distributions of ground-level daily PM_2.5_ concentrations and 10 m wind (black arrows) in Tangshan on 3 December 2016, derived from (**a**) the CAQRA dataset, and the WRF–Chem (**b**) BASE run and (**c**) BLK run. In (**d**), the daily PM_2.5_ concentrations in Tangshan (outlined by the purple rectangle in (**a**–**c**) are displayed). The red contour delineates the boundary of Tangshan.

Regional synoptic patterns not only regulate the PBL structure but also dictate pollutant transport pathways [37,38]. In addition to creating a shallow PBL, Type 5 can bring in warmer, more polluted air masses from the southwest (Figure 13d–f). As a result, daily average PM_2.5_ concentrations in Tangshan exceed 180 μg m^−3^ (Figure 14b). Conversely, Type 1 is associated with dry, clean, and strong northwesterly winds (Figure 13a–c), which promote the horizontal dispersion and dilution of pollutants, reducing Tangshan’s daily average PM_2.5_ concentration to below 30 μg m^−3^ (Figure 14a). For example, on 3 December 2016, Tangshan was under the influence of synoptic Type 5. The simulated daily PM_2.5_ concentration in Tangshan (Figure 15) from the WRF–Chem BASE run was 192.3 µg m^−3^. According to the BLK run, which excludes local emissions, the contribution from regional transport was 103.8 µg m^−3^, accounting for approximately 54% of the total PM_2.5_ concentration (Figure 15d). This demonstrates the significant role of regional transport in contributing to PM_2.5_ pollution in Tangshan under synoptic Type 5.

**Figure 16:**
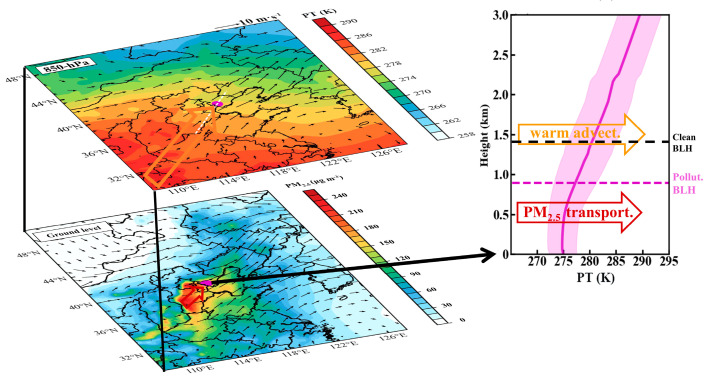
Schematic diagram illustrating the physical mechanisms behind PM_2.5_ pollution events in Tangshan. Key processes include warm advection above PBL, enhanced thermal inversion layer, limited PBL, and the southwesterly transport of pollutants within the PBL.

In summary, the presence of a north–south pressure gradient at the 850 hPa level over North China triggers southwesterly winds that transport pollutants from cities in the adjacent Beijing–Tianjin–Hebei region to Tangshan (Figure 16). Vertically, warm advection in the upper PBL enhances the thermal stability of the lower troposphere, limiting the development of the daytime PBL. The resulting lower BLH restricts the vertical dispersion and dilution of pollutants in Tangshan, leading to the persistent accumulation of near-surface pollutants. These multi–scale meteorological forces play a crucial role in the frequent severe winter PM_2.5_ pollution events in Tangshan. Therefore, to improve regional air quality, coordinated efforts between Tangshan and the Beijing–Tianjin–Hebei region are essential to address their shared air pollution challenges.

## 4. Conclusions

This study highlights the relationship between PM_2.5_ pollution in Tangshan and multi–scale meteorological processes. Both seasonal and day–to–day variations in PM_2.5_ are closely tied to atmospheric thermal stability, with heightened stability in the lower troposphere during winter leading to reduced BLH and severe pollution events. Regional synoptic patterns, particularly the presence of a high–pressure system over the southern North China Plain at the 850 hPa level, drive upper–level warm advection that strengthens the thermal inversion layer, limits PBL development, and limits vertical pollutant dispersion. Prevailing southwesterly winds within PBL further transport pollutants from the Beijing–Tianjin–Hebei region to Tangshan, exacerbating pollution. 

To improve the poor air quality, local governments in Tangshan and neighboring areas should implement targeted emission reduction strategies, particularly during winter. This could include stricter regulations on industrial emissions and improved traffic management in high–pollution areas. Enhancing air quality monitoring and forecasting systems would enable timely warnings and proactive measures to protect public health. Regional cooperation is also crucial to address the transboundary nature of pollution [63], as emission control efforts across the region would have a more significant impact.

Future research should explore long–term changes in meteorological conditions and their effects on PM_2.5_ pollution, including the influence of extreme weather events. Additionally, given the dominance of westerly winds in Northeast Asia [64], studying the long-range transport of air pollutants could offer valuable insights into regional and transboundary air quality management strategies. 

## Data Availability

The ERA5 datasets analyzed can be found in the Science Data Bank (https://cds.climate.copernicus.eu/cdsapp#!/search?type=dataset) (accessed on 8 January 2023), the CAQRA datasets analyzed can be found in the Science Data Bank (https://doi.org/10.11922/sciencedb.00053), the meteorological measurements are provided by the China Meteorological Administration (http://data.cma.cn/) (accessed on 8 January 2023), the PM_2.5_ data are provided by the China National Environmental Monitoring Center (http://datacenter.mee.gov.cn) (accessed on 8 January 2023), and the MEIC datasets are provided by Tsinghua University (http://www.meicmodel.org) (accessed on 8 January 2023).

## Supplementary Materials

The following supporting information can be downloaded at: https://www.mdpi.com/article/10.3390/toxics12090685/s1, Figure S1. Monthly variation of PM_2.5_ concentration (black) and anthropogenic PM_2.5_ emissions (grey) in Tangshan from 2015 to 2019. The anthropogenic PM_2.5_ emissions were derived from MEIC data; Figure S2. Time–height sections of monthly average potential temperature (PT) derived from (a) soundings and (b) ERA5 data at 20:00 LT in Tangshan from 2015 to 2019. The gray dotted lines represent monthly average PM_2.5_ concentration, while the black dotted lines indicate monthly average boundary layer height (BLH) at 20:00 LT; Figure S3. Time-height sections of day-to-day PT variations derived from (a) soundings and (b) ERA5 data at 20:00 LT from 1 December 2016 to 28 February 2017, overlaid with observed daily mean PM_2.5_ concentration; Figure S4. Differences between polluted and clean days in (from left to right) PT, specific humidity (q), wind speed (WS), and wind direction at 700 hPa, 850 hPa, and 925 hPa in Tangshan during the winters of 2015–2019. The data is derived from the ERA5 dataset at 14:00 LT; Figure S5. Eight synoptic pattern types of 850 hPa geopotential height (GH) fields (color shading) identified using the T–PCA method, overlaid with wind vector fields (black arrows). The occurrence frequency (%) is indicated at the bottom of each panel; Table S1. Summary (mean ± std) of the eight identified synoptic types, including 850 hPa potential temperature (PT_850_), wind speed (WS_850_), wind direction (WD_850_), boundary layer height (BLH), and the ground-level PM_2.5_ concentration in Tangshan.
