# Supplementary material for: Multi-Scale Meteorological Impact on PM2.5 Pollution in Tangshan, Northern China
Source: Toxics. 2024 Sep 22;12(9):685. doi: 10.3390/toxics12090685 (PMC11435594; doi:10.3390/toxics12090685)
Supplement: Supplementary file 1 [file toxics-12-00685-s001.zip › toxics-3176224-supplementary.pdf]

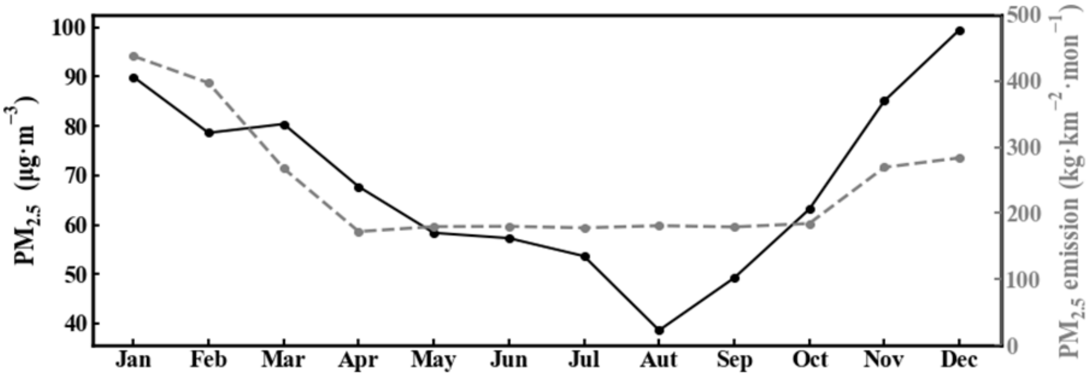

Figure S1. Monthly variation of $PM_{2.5}$ concentration (black) and anthropogenic $PM_{2.5}$ emissions (grey) in Tangshan from 2015 to 2019. The anthropogenic $PM_{2.5}$ emissions were derived from MEIC data.

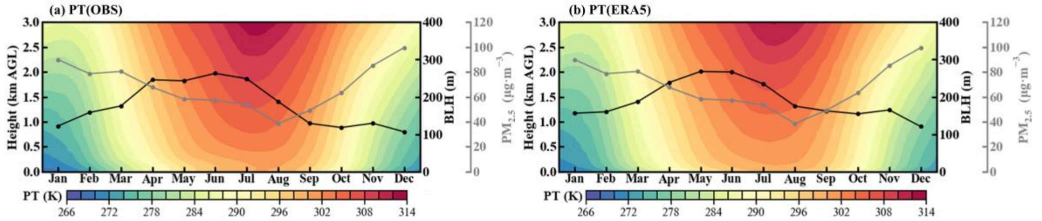

Figure S2. Time-height sections of monthly average potential temperature (PT) derived from (a) soundings and (b) ERA5 data at 20:00 LT in Tangshan from 2015 to 2019. The gray dotted lines represent monthly average $PM_{2.5}$ concentration, while the black dotted lines indicate monthly average boundary layer height (BLH) at 20:00 LT.

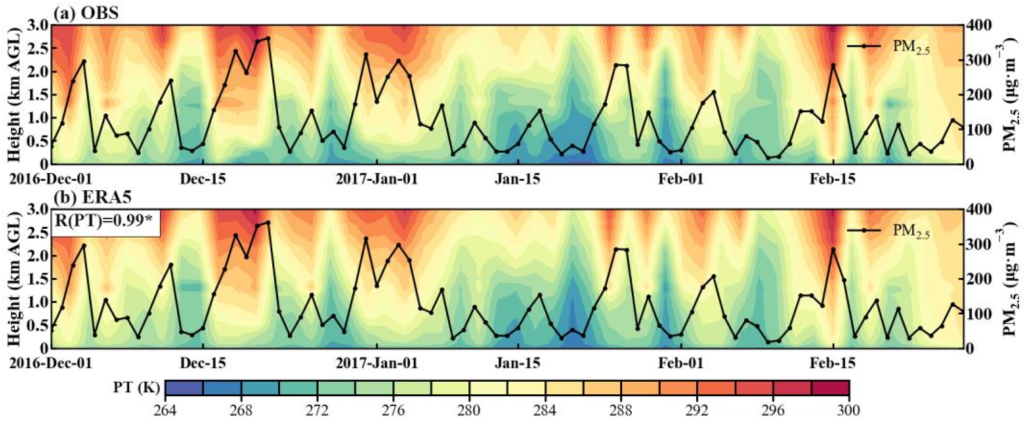

Figure S3. Time-height sections of day-to-day PT variations derived from (a) soundings and (b) ERA5 data at 20:00 LT from 1 December 2016 to 28 February 2017, overlaid with observed daily mean $PM_{2.5}$ concentration.

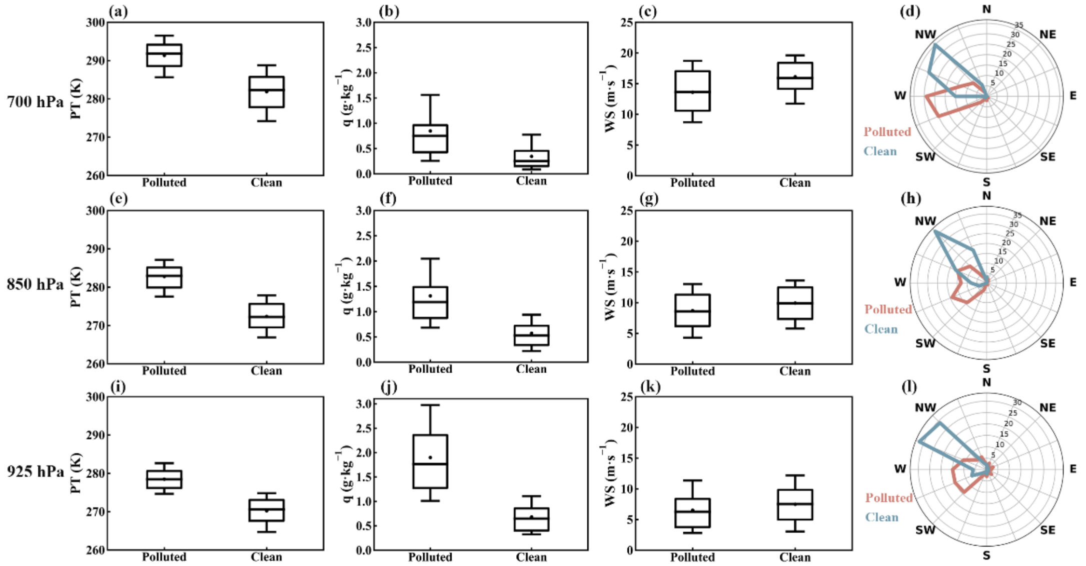

Figure S4. Differences between polluted and clean days in (from left to right) PT, specific humidity (q), wind speed (WS), and wind direction at 700 hPa, 850 hPa, and 925 hPa in Tangshan during the winters of 2015-2019. The data is derived from the ERA5 dataset at 14:00 LT.

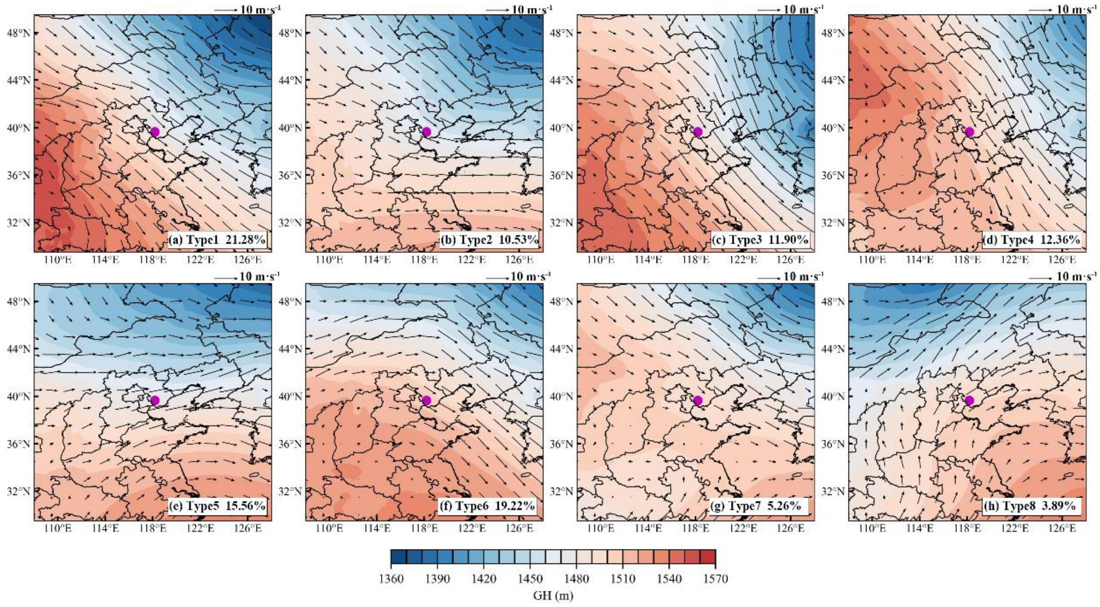

Figure S5. Eight synoptic pattern types of 850 hPa geopotential height (GH) fields (color shading) identified using the T-PCA method, overlaid with wind vector fields (black arrows). The occurrence frequency (%) is indicated at the bottom of each panel.

Table S1. Summary (mean ± std) of the eight identified synoptic types, including 850 hPa potential temperature (PT_{850}), wind speed (WS_{850}), wind direction (WD_{850}), boundary layer height (BLH), and the ground-level $PM_{2.5}$ concentration in Tangshan.

Type	Frequency (%)	PT_{850} (K)	WD_{850} ($m \cdot s^{-1}$)	WS_{850} (°)	BLH (m)	$PM_{2.5}$ ($\mu g\ m^{-3}$)
1	21.28	272.6±5.0	9.1±3.6	311	1391±524	74±64
2	10.53	280.4±3.7	8.5±3.6	296	1024±513	120±73
3	11.90	275.6±4.7	8.2±3.2	324	1286±485	73±40
4	12.36	274.0±5.5	6.7±3.4	330	1305±408	66±50
5	15.56	280.9±4.8	9.2±3.5	258	894±415	128±65
6	19.22	278.8±4.2	6.8±3.6	300	935±317	92±62
7	5.26	279.0±5.4	4.9±4.3	302	904±320	112±81
8	3.89	282.9±4.0	5.9±3.1	234	989±247	129±77
